# Rare Causes of Portal Vein Thrombosis and Occlusion: A Narrative Review

**DOI:** 10.3390/diagnostics16050800

**Published:** 2026-03-08

**Authors:** Lavinia Alice Bălăceanu, Claudia Georgeta Iacobescu, Teodora Burloiu, Marian-Vlad Lăpădat, Ion Dina, Ion Daniel Baboi

**Affiliations:** 1 Clinical Department 1—Medical Semiology, “Carol Davila” University of Medicine and Pharmacy, 020021 Bucharest, Romania; alice.balaceanu@umfcd.ro (L.A.B.); daniel.baboi@umfcd.ro (I.D.B.); 2 Internal Medicine Department, Clinical Emergency Hospital “Sf. Ioan”, 042122 Bucharest, Romania; 3 Gastroenterology Department, Clinical Emergency Hospital “Sf. Ioan”, 042122 Bucharest, Romania; iacobescu_clodi@yahoo.com (C.G.I.); teodora.lupu94@gmail.com (T.B.)

**Keywords:** portal vein thrombosis, portal vein occlusion, non-cirrhotic portal vein thrombosis, post-splenectomy portal vein thrombosis, hepatic portal venous gas

## Abstract

Portal vein thrombosis (PVT) refers specifically to the presence of a thrombus within the main portal vein trunk or its intrahepatic branches. In contrast, portal vein occlusion encompasses a broader spectrum of conditions, including tumor invasion, external compression and disorders that predispose to thrombosis, such as thrombophilia or inflammatory states. Advanced liver disease, particularly cirrhosis, is the most common cause of PVT, primarily due to portal hypertension, altered hemostasis and hemodynamic changes, followed by malignancies and inherited or acquired thrombophilic conditions. In contrast to these common etiologies, our clinical experience has highlighted rare causes of portal vein obstruction associated with typical presentations, which pose diagnostic challenges. Examples include acute PVT during transjugular intrahepatic portosystemic shunt (TIPS) placement and non-thrombotic porto-mesenteric obstruction related to portal venous gas. While these events may appear unexpected, they represent a recognizable group of uncommon causes rather than isolated incidents. PVT can present as an acute or chronic condition: acute thrombosis is characterized by recent thrombus formation and potential intestinal ischemia, whereas chronic thrombosis is associated with long-standing obstruction, cavernous transformation and portal hypertension. This narrative review integrates a comprehensive literature search with clinical experience, with particular emphasis on uncommon etiologies of portal vein obstruction.

## 1. Introduction

Portal vein thrombosis (PVT) is a relatively rare condition in the general population, but its prevalence increases significantly in patients with underlying liver disease, malignancy, or other prothrombotic conditions [1,2,3,4,5]. Traditionally, PVT refers to non-malignant thrombus formation within the portal venous system; however, malignant portal vein thrombosis can also occur through multiple mechanisms beyond direct tumor invasion, including hypercoagulability induced by neoplasms and inflammatory or endothelial changes [6]. For conceptual clarity, portal vein thrombosis (PVT) in this review refers strictly to intraluminal thrombus formation within the portal venous system. In contrast, portal vein occlusion is used as a broader term encompassing thrombotic obstruction, tumor invasion (portal vein tumor thrombosis), extrinsic compression and non-thrombotic causes such as portal venous gas. This distinction is essential for diagnostic and therapeutic decision-making. The most common condition associated with PVT is liver cirrhosis, a chronic liver disease with multiple complications, most of which are driven by increased pressure within the portal system [7,8,9]. The reported prevalence of PVT in cirrhosis varies widely across studies and is influenced by the diagnostic methods employed as well as the stage of cirrhosis, whether compensated or decompensated [10,11,12]. The risk of PVT increases with the progression of liver cirrhosis. While it may be approximately 10% in the early stages, it rises to 17–26% in patients with Child–Pugh B or C stage, particularly among liver transplant candidates [12,13,14,15]. Several additional factors, aside from disease severity, may influence the development of PVT in cirrhotic patients. These include metabolic disorders—particularly nonalcoholic steatohepatitis, now referred to as MASLD (metabolic dysfunction-associated steatotic liver disease)—the presence of large portosystemic collaterals, a history of previous splanchnic thrombosis, splenectomy and partial splenic embolization [12,16,17]. The latter is a minimally invasive procedure used to correct severe thrombocytopenia associated with advanced cirrhosis [16]. Regarding the mechanisms of PVT in cirrhosis, hemodynamic changes in portal blood flow—particularly reduced flow velocity and portal hypertension—play a central role [1,18,19,20]. These hemodynamic alterations are part of Virchow’s triad, which in cirrhosis includes portal venous stasis, a prothrombotic state and endothelial dysfunction, and all of which contribute to thrombus formation [21,22,23]. While chronic liver disease plays a major role in PVT development, malignancies also constitute a significant etiological factor. The neoplasms most frequently associated with PVT are intra-abdominal tumors and metastatic disease [24]. Hepatocellular carcinoma (HCC) represents the most common primary liver malignancy and is frequently complicated by portal vein tumor thrombosis (PVTT), a condition associated with aggressive tumor behavior, poor prognosis and limited therapeutic options [25,26,27,28]. Across various studies, the reported incidence of PVTT inpatients with HCC ranges from approximately 16% to 30%, underscoring its frequent occurrence and significant influence on disease progression and clinical outcomes [25,29,30]. Furthermore, almost 20% of patients have macrovascular invasion at the time of diagnosis [31]. The presence of PVTT is of major clinical and therapeutic importance, given that it precludes curative management of HCC, such as surgical resection or liver transplantation, in the majority of cases [32,33,34]. In addition, patients with HCC and PVTT have a significantly reduced overall survival rate compared to those without vascular invasion [35,36]. Different mechanisms have been proposed to explain the development of PVT in HCC associated with liver cirrhosis, beyond the well-known direct tumor invasion [6]. One such mechanism is the hypercoagulable state induced by malignancy, in which tumor cells produce and release various procoagulant factors and inflammatory mediators [37].

Most available reviews predominantly focus on cirrhosis-related PVT and PVTT in hepatocellular carcinoma. In contrast, rare etiologies and atypical mechanisms of portal vein obstruction are often reported only as isolated case reports or small case series, without integration into a structured diagnostic framework.

In addition to the more common causes of PVT, such as cirrhosis-related PVT and PVTT associated with liver cancer, portal vein occlusion encompasses a heterogeneous group of conditions, including thrombotic disorders, abdominal inflammatory or metabolic states (pancreatitis, diverticulitis, MASLD), abdominal surgery including bariatric procedures, intra-abdominal sepsis (phylephlebitis) and extrinsic compressive processes that impair portal venous flow [17,38,39,40]. Clinically, PVT may present in either an acute or chronic form, depending on the underlying etiology and the duration of the vascular obstruction. From another perspective, portal vein thrombosis can also be considered an important cause of non-cirrhotic, prehepatic portal hypertension, highlighting its relevance beyond cirrhosis-related complications [19].

Apart from these etiologies, we also discuss acute portal vein thrombosis developing during transjugular intrahepatic portosystemic shunt (TIPS) placement, as well as non-thrombotic porto-mesenteric obstruction related to portal venous gas. An unusual scenario involving portal vein thrombosis combined with hepatic infarction in a thrombophilic patient with a history of myocardial infarction further illustrates the spectrum of rare PVT etiologies.

This narrative review integrates a critical appraisal of current evidence on both common and uncommon etiologies of portal vein thrombosis, contextualizes rare clinical scenarios through illustrative cases from our own experience and provides a structured perspective for the evaluation of patients presenting with atypical portal vein obstruction. By doing so, it aims to improve diagnostic accuracy, facilitate recognition of less common forms of PVT and support clinical decision-making in complex cases.

## 2. Materials and Method

A comprehensive literature search was conducted across major databases, including PubMed and Web of Science (Clarivate Analytics), to identify relevant publications related to portal vein thrombosis and portal vein occlusion. The search focused primarily on articles published within the last five years in order to identify the latest evidence, while earlier landmark studies were also considered when relevant to provide historical context and highlight foundational evidence. The search strategy used free text and MeSH terms including “portal vein thrombosis”, “portal vein”, “venous thrombosis” and “vascular occlusion” as well as combinations of keywords such as “portal vein thrombosis”, “portal vein occlusion”, “non-cirrhotic portal vein thrombosis and “portal venous gas”. Boolean operators (AND/OR) were applied to combine terms appropriately. Full-text original research articles, reviews and meta-analyses published in English were included, along with a limited number of case reports and case series, which were considered to provide additional insights into rare clinical presentations. Editorials and non-English publications were excluded. Article selection was based on relevance to the topic, clinical applicability and contribution to the understanding of rare etiologies of portal vein obstruction, as well as to capture both common and uncommon causes of portal vein thrombosis and occlusion. This review was conducted as a narrative synthesis rather than an exhaustive systematic review; therefore, no formal quality assessment or quantitative meta-analysis was performed.

## 3. Overview of the Evidence

For clarity, in this section, we distinguish between portal vein thrombosis (PVT), referring specifically to thrombotic events and portal vein occlusion, which includes thrombotic as well as non-thrombotic causes, such as tumor invasion or external compression.

The comprehensive search of major medical databases yielded more than 2000 publications related to portal vein thrombosis and portal vein occlusion, including over 1000 studies focused on portal vein thrombosis in patients with liver cirrhosis. Relevant articles were selected for inclusion in this narrative review, with attention to clinical applicability and the coverage of both common and rare etiologies.

Published data identifies portal vein thrombosis as a frequent complication in patients with liver cirrhosis, with reported incidence increasing with advancing disease severity, whereas in the general population, it remains a rare condition [11,12,41,42,43]. Multiple risk factors associated with the development and progression of portal vein thrombosis have been described. The main determinants include alterations in the components of Virchow’s triad, namely portal stasis, endothelial injury and a hypercoagulable state [12,44,45,46].

The included studies are heterogeneous, encompassing different populations, study designs and diagnostic methods, which should be considered when interpreting the prevalence and risk factors of PVT.

While liver cirrhosis represents the most frequent underlying condition associated with portal vein thrombosis, a wider range of predisposing factors has been described. These include both local and systemic risk factors, among which cirrhosis constitutes a major but not exclusive contributor. Local risk factors include abdominal malignancies, inflammatory or infectious diseases, surgical interventions and portal hemodynamic alterations (Table 1) [17,35,36,47,48,49]. It should be noted that the relative contribution of each factor varies across studies, reflecting differences in patient populations, study design and diagnostic approaches.

Malignancy is a well-established risk factor for thrombosis at various vascular sites, including the splanchnic veins. Among abdominal malignancies, hepatocellular carcinoma (HCC) and pancreatic neoplasms are particularly associated with portal vein thrombosis, which may represent an occult manifestation of these malignancies [47,48]. In HCC, PVTT correlates with markers of tumor aggressiveness, increased tumor burden, abnormal liver biochemistry, elevated AFP levels and poor patient functional status [6]. Importantly, the patient’s outcome is influenced not only by the presence of PVTT but also by its extent and severity [25]. Conversely, portal vein thrombosis is rarely encountered in colorectal cancer [49]. When present, it usually occurs in the context of advanced disease, characterized by aggressive tumor behavior and vascular invasion, features that are associated with poor prognosis [49,50]. The mechanism of PVT development in abdominal cancer is multifactorial, involving the intrinsic hypercoagulable state induced by malignancy, the release of proinflammatory cytokines by tumor cells that promote thrombogenesis, direct vascular invasion with subsequent endothelial damage and finally chemotherapy and radiotherapy [49].

A recent meta-analysis including almost 27,000 patients with metabolic dysfunction-associated steatotic liver disease (MASLD) reported a prevalence of portal vein thrombosis of 8.5% [17,51]. Moreover, patients with MASLD, particularly those with advanced disease, have shown a significantly increased risk of PVT compared with patients with advanced liver disease of other etiologies [17]. In fact, MASLD has been identified as an independent risk factor for PVT through its pro-inflammatory and pro-oxidative effects.

Pylephlebitis, defined as suppurative thrombosis of the portal venous system, is a rare clinical condition with a low incidence. It typically arises as a complication of any abdominal or pelvic infection within the drainage area of the portal venous system. The most common causes include acute appendicitis and diverticulitis, although pancreatitis and cholangitis can also lead to suppurative portal vein thrombosis [40,52,53]. Overall, it carries significant mortality and early diagnosis requires a high index of suspicion for timely management.

A meta-analysis of six studies including over 5000 patients with liver cirrhosis showed that splanchnic vein thrombosis (SVT) is a recognized complication following splenectomy and splenic artery embolization, procedures commonly performed to manage hypersplenism and portal hypertension [16,54]. Although SVT is well described in this context, data specifically addressing portal vein thrombosis (PVT) remain scarce.

Regarding TIPS, Wan et al. reported that portal vein thrombosis can occur both before and after transjugular intrahepatic portosystemic shunt placement in patients with liver cirrhosis to decompress the portal system, highlighting the need for careful monitoring of portal vein patency in these patients [55].

In patients with liver cirrhosis, splenectomy performed for refractory esophagogastric variceal bleeding has been associated with up to a tenfold increase in the risk of portal vein thrombosis; however, it may also reduce portal hypertension, improve liver function and attenuate hepatic fibrosis [56,57,58]. A study conducted in 45 patients with liver cirrhosis showed that both the MELD score and portal vein diameter were independently associated with PVT, with incidence rates varying from 18.9% to 57.0%, exceeding those observed in non-surgical cirrhotic populations [56].

In addition to local precipitating factors, several systemic conditions have been implicated in the development of portal vein thrombosis (Table 2) [47].

In addition to local factors, several systemic conditions contribute to the risk of PVT, reflecting the multifactorial nature of this condition. Hematological conditions, including myeloproliferative neoplasms and particularly polycythemia vera, are well-established risk factors due to their strong prothrombotic potential [1,47]. Inherited thrombophilic disorders also play a significant role in PVT pathogenesis [47,58]. Exogenous factors, including hormone-based medication, especially estrogen-containing oral contraceptives and hormone replacement therapy, further increase the risk of portal vein thrombosis by enhancing coagulation activity [47,59]. In addition to the aforementioned factors, CMV (cytomegalovirus) infection has been reported as a potential contributor to portal vein thrombosis [60].

## 4. Contextualizing Rare Cases of Portal Vein Thrombosis and Occlusion

In order to enhance the clinical applicability of the reviewed evidence, uncommon etiologies of portal vein thrombosis and portal vein occlusion are contextualized through illustrative cases derived from our institutional experience. Rather than serving as isolated observations, these cases are integrated into the existing literature to highlight diagnostic pitfalls, pathophysiological mechanisms and management implications in atypical clinical scenarios.

Portal vein thrombosis can present acutely with severe abdominal pain and it may lead to mesenteric ischemia if the thrombus extends into the mesenteric veins, a condition associated with an unfavorable outcome [60,61,62]. In contrast, chronic portal vein thrombosis, defined by the presence of a thrombus within the portal vein system for more than 6 months, is often asymptomatic and may be discovered incidentally. However, it can later manifest with complications of portal hypertension, such as splenomegaly, ascites or variceal bleeding [63,64]. In this context, biomarkers associated with serosal inflammation, such as CA125, may be elevated in patients with portal hypertension-related ascites, even in the absence of malignancy and could provide additional diagnostic or prognostic insight [65]. Cavernomatous transformation of the portal vein represents a chronic sequela of portal thrombosis, characterized by the formation of a network of collateral venous channels that bypass the occluded portal vein [66].

Although portal vein thrombosis is most frequently encountered in the setting of advanced liver cirrhosis and malignancy, the clinical spectrum of portal vein obstruction is considerably broader. Rare and atypical etiologies, while individually uncommon, collectively represent a clinically relevant subgroup that poses significant diagnostic and therapeutic challenges. These entities are often underrecognized because they occur outside the classic cirrhosis–malignancy paradigm and may present with nonspecific or unexpected clinical features.

In addition to the literature findings, three illustrative clinical cases from the authors’ experience are presented to exemplify uncommon etiologies of portal vein thrombosis and occlusion. The clinical cases were included for illustrative purposes only and were not intended for statistical analysis.

### 4.1. Portal Vein Thrombosis and TIPS-Related Complications

Transjugular Intrahepatic Portosystemic Shunt (TIPS) represents an established therapeutic procedure performed in cases of refractory ascites and uncontrolled variceal bleeding with the aim of decompressing the portal venous system [67]. Documented short-term or intraprocedural complications occur in less than 5% of cases and include intraperitoneal bleeding, arterial injury, hemobilia, sepsis, TIPS dysfunction or thrombosis, while the most frequent long-term complication is worsening hepatic encephalopathy [67,68]. Although portal vein thrombosis was historically regarded as a contraindication to TIPS placement, technical advances and increased operator experience have largely overcome this limitation. Shunt dysfunction may occur due to de novo thrombosis after the procedure or progression of a pre-existing thrombus [69,70]. Moreover, while TIPS is an effective treatment for complications of portal hypertension, it may paradoxically predispose to portal vein thrombosis through endothelial injury, altered flow dynamics or technical complications [71]. Consequently, TIPS and portal vein thrombosis are now considered to have a bidirectional relationship: portal vein thrombosis may complicate TIPS placement or function, but conversely, TIPS can facilitate portal vein recanalization and restore hepatopetal flow, thereby promoting thrombus resolution in selected cases [72].

**Figure 1:**
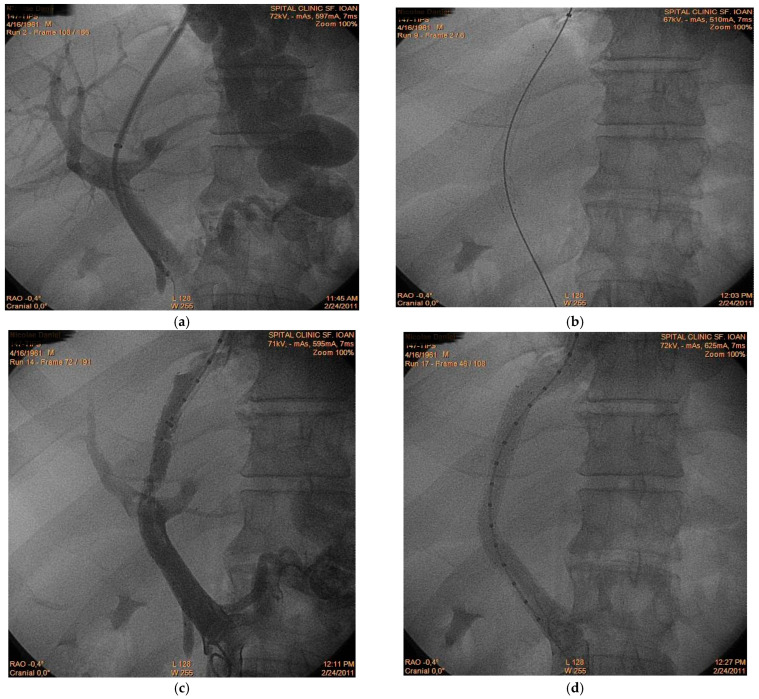
TIPS-related acute portal vein thrombosis (**a**) Initial portal venography demonstrating intrahepatic tract creation; (**b**) First stent placement; (**c**) Intraprocedural acute thrombus formation between portal and suprahepatic vein; (**d**) Post-stent restoration of flow following second stent placement.

A 56-year-old male patient with alcoholic liver cirrhosis was admitted for acute variceal bleeding refractory to endoscopic therapy. Within the following 2 months, the patient experienced two episodes of rebleeding; therefore, the hemorrhage was considered refractory to standard therapy. The decision was made to perform transjugular intrahepatic portosystemic shunt (TIPS) placement as secondary prophylaxis. However, immediately after intrahepatic tract creation (Figure 1a), acute thrombus formation was observed within the main portal vein (Figure 1b,c), suggesting intraprocedural de novo thrombosis rather than embolization of a pre-existing clot. Prompt placement of a second stent restored the portal flow (Figure 1d) and the patient had a favorable outcome. This case illustrates a rare but clinically significant phenomenon: rapid intraprocedural portal vein thrombosis. While shunt dysfunction and delayed thrombosis are recognized complications, acute thrombus formation during tract creation is scarcely reported. The observation supports the hypothesis that endothelial trauma and abrupt hemodynamic changes may trigger immediate thrombogenesis in a prothrombotic cirrhotic milieu. Awareness of this possibility is essential, as rapid recognition and mechanical correction may prevent shunt failure and early clinical deterioration.

### 4.2. Non-Thrombotic Portal Vein Occlusion and Portal Venous Gas

Hepatic portal venous gas (HPVG) represents an abnormal accumulation of gas within the portalvenous system, often reflecting a severe underlying condition. In the majority of cases, HPVG is associated with a poor prognosis. Data in the literature remains limited, with only a few case reports and small case series documenting this phenomenon. Portal venous gas is traditionally considered a radiologic sign of severe intra-abdominal pathology, including bowel ischemia, necrotizing enterocolitis or intra-abdominal sepsis [73]. However, HPVG may also occur in iatrogenic settings, such as following endoscopic procedures or abdominal surgery [74,75]. In these cases, mechanical obstruction, increased intraluminal pressure or inflammatory injury to the venous wall may impair portal flow without true thrombosis. Differentiating thrombotic from non-thrombotic obstruction is critical, as management strategies differ substantially.

**Figure 2:**
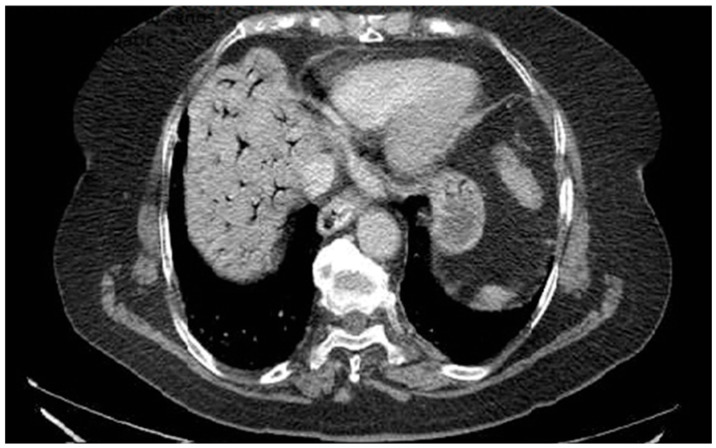
Portal Vein Occlusion due to portal venous gas.

A 76-year-old female patient with no significant past medical history presented with severe upper abdominal pain of more than two days’ duration. On admission, she was hemodynamically unstable, with rapid and acute clinical deterioration of general status and signs ofperitoneal irritation at abdominal examination. Biological markers, including inflammatory parameters and liver function tests, were within normal limits, except for elevated D-dimer levels. Contrast-enhanced computed tomography (CT) revealed extensive gas within the intrahepatic portal venous branches, without evidence of pneumoperitoneum or overt gastrointestinal perforation (Figure 2). The patient was admitted to theintensive care unit for continuous monitoring and intensive resuscitative therapy. Despite supportive management, the outcome was unfavorable, with clinical deterioration precluding surgical intervention. The patient died within 12 h of admission. No necropsy was performed and the underlying cause of portal venous gas remained undetermined. This case highlights several clinically relevant aspects. First, portal venous gas may occur in the absence of overt radiologic signs of perforation or advanced laboratory abnormalities, potentially delaying etiologic clarification. Second, HPVG should be interpreted as a radiologic sign rather than a diagnosis itself; it reflects an underlying pathophysiological process that may range from reversible mucosal injury to catastrophic mesenteric ischemia. Third, from a diagnostic perspective, the absence of visible thrombus emphasizes the distinction between portal vein thrombosis and non-thrombotic portal vein occlusion. Recognizing this difference is essential, as anticoagulation—central to thrombotic PVT management—may not address the primary pathology in HPVG and could even be inappropriate in certain contexts. Therefore, HPVG requires urgent etiologic assessment, multidisciplinary evaluation and individualized therapeutic decision-making.

### 4.3. Thrombophilia-Associated Portal Vein Thrombosis

Thrombophilia encompasses a heterogeneous group of disorders characterized by an imbalance in the regulation of coagulation and fibrinolysis, leading to an increased tendency for thrombosis [76]. The risk of developing thrombophilia is related to both inherited and environmental factors [76]. Systemic congenital and acquired thrombophilia, such as Factor V Leiden mutation, antithrombin III deficiency and prothrombin G20210A mutation, have been reported in patients with portal vein thrombosis. Although individually uncommon, these inherited prothrombotic conditions can significantly contribute to the overall thrombotic risk, particularly in patients with non-cirrhotic portal vein thrombosis [1,38,77]. Regarding protein C and protein S deficiencies, evidence regarding their thrombophilic role remains inconclusive, largely because many studies included patients with chronic liver disease and the levels of these natural anticoagulants can be influenced by anticoagulant therapy or by the acute thrombotic event itself [38]. Prothrombin G20210A and Factor V Leiden mutation have been associated with an increased risk of portal vein thrombosis in patients with cirrhosis [78,79]. Patients with cirrhosis who carry these inherited thrombophilic mutations have approximately a two fold higher risk of developing PVT compared to those without such mutations [78]. Overall, the prevalence of both acquired and inherited thrombophilia in cirrhotic patients with PVT appears low, supporting targeted, individualized testing rather than universal screening [78,79]. The following case illustrates the clinical impact of thrombophilia-associated portal vein thrombosis in a patient with underlying cirrhosis and Factor V Leiden mutation, highlighting how inherited prothrombotic conditions can lead to severe complications such as hepatic infarction.

**Figure 3:**
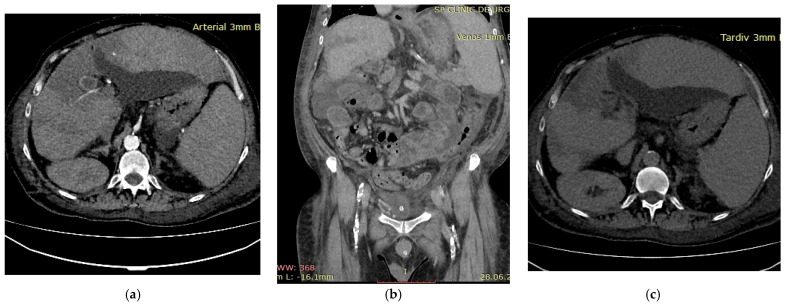
Multiphasic CT imaging (**a**) Arterial phase imaging showed reduced enhancement of segmental hepatic arterial branches; (**b**) Portal venous phase showed portal vein thrombosis; (**c**) Delayed venous phase showed patent hepatic veins.

A 52-year-old male patient with decompensated alcoholic liver cirrhosis was admitted with progressive liver failure. His past medical history was notable for untreated diabetes and inherited thrombophilia due to Factor V Leiden mutation, diagnosed 20 years earlier following an acute myocardial infarction. Biological parameters indicated severe hepatic failure, hyperglycemia and elevated glycated hemoglobin, reflecting poor metabolic control and concomitant renal dysfunction. Multiphasic contrast-enhanced computed tomography revealed thrombosis of the portal venous system during the portal venous phase (Figure 3b). Arterial-phase imaging revealed reduced enhancement of segmental hepatic arterial branches supplying a centrally localized infarcted area (Figure 3a), while delayed images confirmed patency of the hepatic veins (Figure 3c), thereby excluding hepatic venous outflow obstruction. Anticoagulant therapy with low molecular weight heparin (LMWH) at a therapeutic dose, adjusted for renal function, was initiated, along with general measures to support organ function. The patient’s general condition deteriorated rapidly and he ultimately died. This case underscores several important clinical considerations. First, inherited thrombophilia may act as a “second hit” in cirrhotic patients, amplifying the prothrombotic milieu already associated with portal hypertension and altered hemostasis. Second, the occurrence of hepatic infarction in cirrhosis is uncommon due to the liver’s dual blood supply; therefore, its presence should prompt evaluation for combined vascular compromise. Third, this case illustrates that a thrombotic burden disproportionate to the severity of liver disease may justify targeted thrombophilia testing, particularly in younger patients or in those with thrombotic events affecting multiple vascular territories. Integrating clinical context, imaging findings and individualized laboratory assessment is essential for risk stratification and therapeutic decision-making in such complex presentations.

## 5. Conclusions

Portal vein thrombosis and portal vein occlusion represent complex vascular disorders requiring individualized diagnostic and therapeutic strategies. While cirrhosis-related and malignancy-associated forms remain predominant, clinicians should maintain a high index of suspicion for rare etiologies in patients presenting with atypical clinical or imaging features. Early differentiation between thrombotic and non-thrombotic portal obstruction is essential, as management strategies differ substantially. Comprehensive imaging assessment—including multiphasic contrast-enhanced CT or MRI—should be performed in all atypical cases to evaluate arterial inflow, portal venous patency and hepatic venous outflow. Targeted thrombophilia testing should be considered in non-cirrhotic patients, in those with recurrent or extensive thrombosis or when the thrombotic burden appears disproportionate to liver disease severity. Routine universal screening in all cirrhotic patients, however, is not supported by current evidence. Future research should aim to establish standardized diagnostic algorithms for rare forms of portal vein obstruction, clarify the role of inherited thrombophilia in cirrhosis-associated PVT, particularly in patients presenting with concomitant thrombotic events in other vascular territories and define optimal anticoagulation strategies in complex dual-inflow thrombosis. Improved recognition of atypical and rare presentations may enhance diagnostic accuracy, guide therapeutic decision-making and ultimately improve patient outcomes.

## Figures and Tables

**Table 1:** Local risk factors for portal vein thrombosis.

Solid Abdominal Cancer	Inflammatory/Infectious Diseases	Injury of PVS *	Cirrhosis
HCC	Neonatal omphalitis	Splenectomy	Splenectomy
Pancreas	Diverticulitis	Liver transplantation	TIPS *
Colorectal	Appendicitis	Colectomy	Surgical Portosystemic shunting
Breast	IBD	Abdominal trauma	Previous variceal ligation
Bladder	Cholecystitis		Large esophageal varices
Gastric	MASLD		
	Pancreatitis		

* PVS—portal venous system. * TIPS—Transjugular Intrahepatic Portosystemic Shunt.

**Table 2:** General risk factors for portal vein thrombosis.

Hematological Diseases	Autoimmune Conditions	Inherited Thrombophilia	Medications	Pregnancy
Myeloproliferative neoplasms	Behcet’s disease	Factor V Leiden mutation	Hormonal Replacement Therapy	
Paroxysmal nocturnal hemoglobinuria	Antiphospholipid syndrome	Antithrombin deficiency	Estrogen-derived oral contraceptives	
		Protein C deficiency		
		Protein S deficiency		

## Data Availability

Data is contained within the article; the presented images are part of the Gastroenterology Department’s collection.

## Abbreviations

The following abbreviations are used in this manuscript:

CMV	Cytomegalovirus
SVT	Splanchnic vein thrombosis
HPVG	Hepatic Portal Venous Gas
NAFLD	Non-alcoholic liver disease
MASLD	Metabolic dysfunction-associated steatotic liver disease
PVS	Portal venous system

## References

[1] Boccatonda A., Gentilini S., Zanata E., Simion C., Serra C., Simioni P., Piscaglia F., Campello E., Ageno W. (2024). Portal Vein Thrombosis: State-of-the-Art Review. J. Clin. Med..

[2] De Franchis R., Bosch J., Garcia-Tsao G., Reiberger T., Ripoll C. (2022). Baveno VII-Renewing consensus in portal hypertension. J. Hepatol..

[3] Elkrief L., Hernandez-Gea V., Senzolo M., Albillos A., Baiges A., Berzigotti A., Bureau C., Murad S.D., De Gottardi A., Durand F. (2024). Portal vein thrombosis: Diagnosis, management, and endpoints for future clinical studies. Lancet Gastroenterol. Hepatol..

[4] Yang Z., Zhao Y., Chen H., Zhang H., Tan M., Li X., Tao L., Zhao H. (2025). Portal Vein Thrombosis in Liver Cirrhosis: A Review of Risk Factors and Predictive Indicators. J. Clin. Transl. Hepatol..

[5] Prakash S., Bies J., Hassan M., Mares A., Didia S.C. (2023). Portal vein thrombosis in cirrhosis: A literature review. Front. Med..

[6] Khan A.R., Wei X., Xu X. (2021). Portal Vein Tumor Thrombosis and Hepatocellular Carcinoma—The Changing Tides. J. Hepatocell. Carcinoma.

[7] Ginès P., Krag A., Abraldes J.G., Solà E., Fabrellas N., Kamath P.S. (2021). Liver cirrhosis. Lancet.

[8] D’Amico G., Garcia-Tsao G., Pagliaro L. (2006). Natural history and prognostic indicators of survival in cirrhosis: A systematic review of 118 studies. J. Hepatol..

[9] Shukla A., Giri S. (2022). Portal Vein Thrombosis in Cirrhosis. J. Clin. Exp. Hepatol..

[10] Odriozola A., Puente Á., Cuadrado A., Rivas C., Anton Á., González F.J., Pellón R., Fábrega E., Crespo J., Fortea J.I. (2022). Portal Vein Thrombosis in the Setting of Cirrhosis: A Comprehensive Review. J. Clin. Med..

[11] Pan J., Wang L., Gao F., An Y., Yin Y., Guo X., Nery F.G., Yoshida E.M., Qi X. (2022). Epidemiology of portal vein thrombosis in liver cirrhosis: A systematic review and meta-analysis. Eur. J. Intern. Med..

[12] Senzolo M., Garcia-Tsao G., García-Pagán J.C. (2021). Current knowledge and management of portal vein thrombosis in cirrhosis. J. Hepatol..

[13] Xian J., Tang Y., Shao H., Wang X., Zhang M., Xing T. (2021). Effect of portal vein thrombosis on the prognosis of patients with cirrhosis without a liver transplant: A systematic review and meta-analysis. Medicine.

[14] Turon F., Driever E.G., Baiges A., Cerda E., García-Criado Á., Gilabert R., Bru C., Berzigotti A., Nuñez I., Orts L. (2021). Predicting portal thrombosis in cirrhosis: A prospective study of clinical, ultrasonographic and hemostatic factors. J. Hepatol..

[15] Zuo H.W., Sha Q.M., Sun J., Cai Z.H., Xu H.W., Liu H. (2021). Risk factors of portal vein thrombosis in cirrhotic patients with esophageal varices. J. Clin. Hepatol..

[16] Omer S., Zara O., Iacobescu C., Dina I. (2014). Partial Splenic Embolization for Hypersplenism in Cirrhotic Patients. A Case Series. J. Gastrointest. Liver Dis..

[17] Stupia R., Lombardi R., Cattazzo F., Zoncapè M., Mantovani A., De Marco L., Mantovani A., Fracanzani A.L., Sacerdoti D., Dalbeni A. (2023). Prevalence of portal vein thrombosis in non-alcoholic fatty liver disease: A meta-analysis of observational studies. J. Thromb. Thrombolysis.

[18] Yan Y., Xiong Z., Wang X., Yang L., Zheng T., Luo X. (2022). A novel potential mechanism for the development of portal vein thrombosis in cirrhosis based on portal hemodynamics. Insights Into Imaging.

[19] Patel M., Hunt C., VanWagner L. (2023). Recent Approaches in Portal Hypertension Involving Risk Stratification and Medical Management. Gastroenterol. Hepatol..

[20] He W.L., Yan S., Lu J.J., Chen L., Wu J.Z. (2025). Current clinical research status and future treatment directions for liver cirrhosis combined with portal vein thrombosis. World J. Hepatol..

[21] Li J., Wang Q., Yang M., Sun X. (2022). Metabolic Disorders and Risk of Portal Vein Thrombosis in Liver Cirrhosis: A Systematic Review and Meta-Analysis. Turk. J. Gastroenterol..

[22] Anton A., Campreciós G., Pérez-Campuzano V., Orts L., García-Pagán J.C., Hernández-Gea V. (2022). The Pathophysiology of Portal Vein Thrombosis in Cirrhosis: Getting Deeper into Virchow’s Triad. J. Clin. Med..

[23] Caiano L.M., Riva N., Carrier M., Gatt A., Ageno W. (2021). Treatment of portal vein thrombosis: An updated narrative review. Minerva Medica.

[24] García-Villa A., Criado-Álvarez J.J., Carnevali M., Aramberri M., Font C., Díaz-Pedroche C. (2023). Cancer-associated splanchnic vein thrombosis: Clinical features upon diagnosis and short-term outcomes. Thromb. Res..

[25] Abdelhamed W., Shousha H., El-Kassas M. (2024). Portal vein tumor thrombosis in hepatocellular carcinoma patients: Is it the end?. Liver Res..

[26] Sung H., Ferlay J., Siegel R.L., Laversanne M., Soerjomataram I., Jemal A., Bray F. (2021). Global Cancer Statistics 2020: GLOBOCAN Estimates of Incidence and Mortality Worldwide for 36 Cancers in 185 Countries. CA Cancer J. Clin..

[27] Reig M., Forner A., Rimola J., Ferrer-Fàbrega J., Burrel M., Garcia-Criado Á., Kelley R.K., Galle P.R., Mazzaferro V., Salem R. (2022). BCLC strategy for prognosis prediction and treatment recommendation: The 2022 update. J. Hepatol..

[28] Giri S., Singh A., Das S., Strubchevska K., Tripathy T., Patel R.K., Kozyk M., Roy A. (2024). Efficacy and safety of transjugular intrahepatic portosystemic shunt in patients with hepatocellular carcinoma—A systematic review and meta-analysis. Indian J. Gastroenterol..

[29] Hanafy A.S., Tharwat E.E. (2021). Differentiation of malignant from non-malignant portal vein thrombosis in liver cirrhosis: The challenging dilemma. Egypt. Liver J..

[30] Carr B.I., Guerra V., Ince V., Isik B., Yilmaz S. (2023). Discordance among aggressiveness characteristics of hepatocellular carcinoma: Portal vein thrombosis and multifocality, related to tumor size, but not to serum alpha-fetoprotein level. Liver Res..

[31] Qadan M., Kothary N., Sangro B., Palta M. (2020). The treatment of hepatocellular carcinoma with portal vein tumor thrombosis. Am. Soc. Clin. Oncol. Educ. Book.

[32] Liu B., Grindrod N., Meyers B.M., Freiburger S., Boldt G., Malik A., Jairam M.P., Brahmania M., Leite L.C., Simone C.B. (2023). Treatment modalities to manage hepatocellular carcinoma patients with portal vein thrombosis: A systematic review and meta-analysis. Ann. Palliat. Med..

[33] Singal A.G., Zhang E., Narasimman M., Rich N.E., Waljee A.K., Hoshida Y., Yang J.D., Reig M., Cabibbo G., Nahon P. (2022). HCC surveillance improves early detection, curative treatment receipt, and survival in patients with cirrhosis: A meta-analysis. J. Hepatol..

[34] Pinter M., Fulgenzi C.A.M., Pinato D.J., Scheiner B. (2025). Systemic treatment in patients with hepatocellular carcinoma and advanced liver dysfunction. Gut.

[35] Siddiqui M.T.U., Fareed G., Khan M.R., Riaz A., Hamid S.S. (2023). Portal vein thrombosis in patients with hepatocellular carcinoma and early cirrhosis—Prevalence and risk factors. ecancermedicalscience.

[36] Cheng S., Wei X., Shi J., Guo W., Feng S., Zhai J., Huang B. (2020). A multidisciplinary team approach to the management of patients with hepatocellular carcinoma with portal vein tumor thrombus. Oncologist.

[37] Zanetto A., Campello E., Pelizzaro F., Farinati F., Burra P., Simioni P., Senzolo M. (2022). Haemostatic alterations in patients with cirrhosis and hepatocellular carcinoma: Laboratory evidence and clinical implications. Liver Int..

[38] Gil-Lopez F., Rios-Olais F.A., Mercado L.A., Harnois D.M. (2025). Portal Vein Thrombosis in Patients Without Cirrhosis: Current Practical Approaches and Treatment Strategies. Diagnostics.

[39] Chan W.K., Chuah K.H., Rajaram R.B., Lim L.L., Ratnasingam J., Vethakkan S.R. (2023). Metabolic Dysfunction-Associated Steatotic Liver Disease (MASLD): A State-of-the-Art Review. J. Obes. Metab. Syndr..

[40] Jevtic D., Gavrancic T., Pantic I., Nordin T., Nordstrom C.W., Antic M., Pantic N., Kaljevic M., Joksimovic B., Jovanovic M. (2022). Suppurative Thrombosis of the Portal Vein (Pylephlebits): A Systematic Review of Literature. J. Clin. Med..

[41] Hilscher M.B., Wysokinski W.E., Andrews J.C., Simonetto D.A., Law R.J., Kamath P.S. (2024). Portal Vein Thrombosis in the Setting of Cirrhosis: Evaluation and Management Strategies. Gastroenterology.

[42] Davis J.P.E., Lim J.K., Francis F.F., Ahn J. (2025). AGA Clinical Practice Update on Management of Portal Vein Thrombosis in Patients with Cirrhosis: Expert Review. Gastroenterology.

[43] Zanetto A., Campello E., Burra P., Senzolo M., Simioni P. (2023). Increased platelet ratio in patients with decompensated cirrhosis indicates a higher risk of portal vein thrombosis. Liver Int..

[44] Zanetto A., Campello E., Senzolo M., Simioni P. (2024). The evolving knowledge on primary hemostasis in patients with cirrhosis: A comprehensive review. Hepatology.

[45] Balaceanu L.A., Dina I.D. (2024). D-dimers in advanced cirrhosis: Useful biomarker or not?. Am. J. Med. Sci..

[46] Aiza-Haddad I., Cisneros-Garza L.E., Morales-Gutiérrez O., Malé-Velázquez R., Rizo-Robles M.T., Alvarado-Reyes R., Barrientos-Quintanilla L.A., Betancourt-Sánchez F., Cerda-Reyes E., Contreras-Omaña R. (2024). Guidelines for the management of coagulation disorders in patients with cirrhosis. Rev. Gastroenterol. México (Engl. Ed.).

[47] European Association for the Study of the Liver (EASL) (2026). EASL Clinical Practice Guidelines on vascular diseases of the liver. J. Hepatol..

[48] Muscat-Baron L., Borg A.L., Attard L.M., Gatt A., Riva N. (2023). Cancer-Associated Abdominal Vein Thrombosis. Cancers.

[49] Shah D. (2020). Diagnosis of portal vein tumor thrombosis in colorectal carcinoma in fluorodeoxyglucose positron emission tomography—Computed tomography scan and its clinical implication. World J. Nucl. Med..

[50] Vrabie C., Ceausu M., Petrescu A., Waller M., Dina I. (2008). The usefulness of immunohistochemistry in sporadic colorectal cancer. Romanian J. Morphol. Embryol..

[51] Boccatonda A., Andreetto L., D’Ardes D., Cocco G., Rossi I., Vicari S., Schiavone C., Cipollone F., Guagnano M.T. (2023). From NAFLD to MAFLD: Definition, Pathophysiological Basis and Cardiovascular Implications. Biomedicines.

[52] Fusaro L., Di Bella S., Martingano P., Crocè L.S., Giuffrè M. (2023). Pylephlebitis: A Systematic Review on Etiology, Diagnosis, and Treatment of Infective Portal Vein Thrombosis. Diagnostics.

[53] Nasir S., Chambers E., Wojkiewicz S. (2022). Pylephlebitis with Splenic and Mesenteric Vein Thrombosis in a Patient with Diverticulitis. Cureus.

[54] Wu Y., Li H., Zhang T., Bai Z., Xu X., Sandri G.B.L., Wang L., Qi X. (2021). Splanchnic Vein Thrombosis in Liver Cirrhosis After Splenectomy or Splenic Artery Embolization: A Systematic Review and Meta-Analysis. Adv. Ther..

[55] Wan Y.-M., Li Y.-H., Wu H.-M., Xu Z.-Y., Xu Y., Yang L.-H., Wu X.-N., Yang J.-H. (2017). Portal vein thrombosis before and after transjugular intrahepatic portosystemic shunt placement: An observational study (STROBE compliant). Medicine.

[56] Li T., Wang L.L., Li Y.P., Gan J., Wei X.S., Mao X.R., Li J.F. (2024). Predictors of portal vein thrombosis after splenectomy in patients with cirrhosis. World J. Hepatol..

[57] Liang Q.S., Xie J.G., Yu C., Feng Z., Ma J., Zhang Y., Wang D., Lu J., Zhuang R., Yin J. (2021). Splenectomy improves liver fibrosis via TNFSF14 (LIGHT) through the JNK/TGF-β1 signaling pathway. Exp. Mol. Med..

[58] Giri S., Angadi S., Varghese J., Sundaram S., Bhrugumalla S. (2023). Prothrombotic states in portal vein thrombosis and Budd-Chiari syndrome in India: A systematic review and meta-analysis. Indian J. Gastroenterol..

[59] LaVasseur C., Neukam S., Kartika T., Bannow B.S., Shatzel J., DeLoughery T.G. (2022). Hormonal therapies and venous thrombosis: Considerations for prevention and management. Res. Pract. Thromb. Haemost..

[60] De Broucker C., Plessier A., Ollivier-Hourmand I., Dharancy S., Bureau C., Cervoni J.-P., Sogni P., Goria O., Corcos O., Sartoris R. (2022). Multicenter study on recent portal venous system thrombosis associated with cytomegalovirus disease. J. Hepatol..

[61] Kumari D. (2025). Acute Mesenteric and Portal Vein Thrombosis: Etiology, Diagnosis, and Interventional Management. Tech. Vasc. Interv. Radiol..

[62] Lorenz J., Kwak D.H., Martin L., Kesselman A., Hofmann L.V., Yu Q., Youssef S., Ciolek P., Ahmed O. (2025). Endovascular management of noncirrhotic acute portomesenteric venous thrombosis. J. Vasc. Interv. Radiol..

[63] Tong W.L., Gadani S. (2025). Chronic Portal Vein Thrombosis: Diagnosis, Management, and Review of the Literature. Tech. Vasc. Interv. Radiol..

[64] Northup P.G., Garcia-Pagan J.C., Garcia-Tsao G., Intagliata N.M., Superina R.A., Roberts L.N., Lisman T., Valla D.C. (2021). Vascular Liver disorders, portal vein thrombosis, and procedural bleeding in patients with Liver disease: 2020 practice guidance by the American Association for the Study of Liver Diseases. Hepatology.

[65] Bălăceanu L.A., Grigore C., Dina I., Gurău C.-D., Mihai M.M., Bălăceanu-Gurău B. (2025). CA125 as a Potential Biomarker in Non-Malignant Serous Effusions: Diagnostic and Prognostic Considerations. J. Clin. Med..

[66] Layton B.M., Lapsia S.K. (2023). The Portal Vein: A Comprehensive Review. Radiographics.

[67] Lee E.W., Eghtesad B., Garcia-Tsao G., Haskal Z.J., Hernandez-Gea V., Jalaeian H., Kalva S.P., Mohanty A., Thabut D., Abraldes J.G. (2024). AASLD Practice Guidance on the use of TIPS, variceal embolization, and retrograde transvenous obliteration in the management of variceal hemorrhage. Hepatology.

[68] Bureau C., Thabut D., Jezequel C., Archambeaud I., D’Alteroche L., Dharancy S., Borentain P., Oberti F., Plessier A., De Ledinghen V. (2021). The use of rifaximin in the prevention of overt hepatic encephalopathy after transjugular intrahepatic portosystemic shunt: A randomized controlled trial. Ann. Intern. Med..

[69] Larrue H., D’Amico G., Olivas P., Lv Y., Bucsics T., Rudler M., Sauerbruch T., Hernandez-Gea V., Han G., Reiberger T. (2023). TIPS prevents further decompensation and improves survival in patients with cirrhosis and portal hypertension in an individual patient data meta-analysis. J. Hepatol..

[70] Lv Y., Fan D., Han G. (2023). Transjugular intrahepatic portosystemic shunt for portal hypertension: 30 years experience from China. Liver Int..

[71] Zhang J., Nie Q., He B., Ma B., Fan X., Liu P., Ye Z. (2025). Transjugular intrahepatic portosystemic shunt for the treatment of portal hypertension complications: A single-center retrospective cohort. JVS-Vasc. Insights.

[72] Molvar C., Amin P. (2021). Portal Vein Thrombosis in Cirrhosis: Interventional Treatment Options. Curr. Gastroenterol. Rep..

[73] Han Z., Qiu B., Zhu Z. (2024). Portal and Mesenteric Venous Gas Due to Necrotizing Enteritis. Indian J. Surg..

[74] Suzuki S., Takeuchi Y., Ishihara R., Kawakami H. (2019). Hepatic Portal Venous Gas Following Colonic Endoscopic Submucosal Dissection. Intern. Med..

[75] Kawakami H., Ban T., Kubota Y. (2018). Iatrogenic hepatic portal venous gas following balloon endoscopy in a patient with hepatico-jejunostomy stricture. Dig. Endosc..

[76] Altwayan R., Tombuloglu H., Alhamid G., Karagoz A., Alshammari T., Alsaeed M., Al-Hariri M., Rabaan A., Unver T. (2025). Comprehensive review of thrombophilia: Pathophysiology, prevalence, risk factors, and molecular diagnosis. Transfus. Clin. Biol..

[77] Gioia S., Riggio O., Nardelli S., Ridola L., Marzano C. (2023). Clinical outcomes and prognostic factors in non-cirrhotic non-neoplastic patients with portal vein thrombosis: A single-centre experience. Dig. Liver Dis..

[78] Ma S.D., Wang J., Bezinover D., Kadry Z., Northup P.G., Stine J.G. (2019). Inherited thrombophilia and portal vein thrombosis in cirrhosis: A systematic review and meta-analysis. Res. Pract. Thromb. Haemost..

[79] Fortea J.I., Carrera I.G., Puente Á., Cuadrado A., Huelin P., Tato C.Á., Fernández P.Á., Montes M.D.R.P., Céspedes J.N., López A.B. (2020). Portal thrombosis in cirrhosis: Role of thrombophilicdisorders. J. Clin. Med..

